# Real-Time Ultrasound/MRI Fusion for Suprasacral Parallel Shift Approach to Lumbosacral Plexus Blockade and Analysis of Injectate Spread: An Exploratory Randomized Controlled Trial

**DOI:** 10.1155/2017/1873209

**Published:** 2017-03-15

**Authors:** Jennie Maria Christin Strid, Erik Morre Pedersen, Sinan Naseer Hussain Al-Karradi, Mathias Alrø Fichtner Bendtsen, Siska Bjørn, Mette Dam, Morten Daugaard, Martin Sejr Hansen, Katrine Danker Linnet, Jens Børglum, Kjeld Søballe, Thomas Fichtner Bendtsen

**Affiliations:** ^1^Department of Anesthesiology and Intensive Care, Aarhus University Hospital, Nørrebrogade 44, 8000 Aarhus C, Denmark; ^2^Department of Radiology, Aarhus University Hospital, Nørrebrogade 44, 8000 Aarhus C, Denmark; ^3^Department of Biomedicine, Faculty of Health, Aarhus University, Vennelyst Boulevard 4, 8000 Aarhus C, Denmark; ^4^Medical Science, Faculty of Health, Aarhus University, Nordre Ringgade 1, 8000 Aarhus C, Denmark; ^5^Department of Anesthesiology and Intensive Care Medicine, Zealand University Hospital, University of Copenhagen, Sygehusvej 10, 4000 Roskilde, Denmark; ^6^Department of Anesthesiology, Regional Hospital of Viborg, Heibergs Alle 4, 8800 Viborg, Denmark; ^7^Department of Anesthesiology and Intensive Care Medicine, Slagelse Hospital, Ingemannsvej 18, 4200 Slagelse, Denmark; ^8^Department of Orthopedic Surgery, Aarhus University Hospital, Tage-Hansens Gade 2, 8000 Aarhus C, Denmark

## Abstract

Fused real-time ultrasound and magnetic resonance imaging (MRI) may be used to improve the accuracy of advanced image guided procedures. However, its use in regional anesthesia is practically nonexistent. In this randomized controlled crossover trial, we aim to explore effectiveness, procedure-related outcomes, injectate spread analyzed by MRI, and safety of ultrasound/MRI fusion versus ultrasound guided Suprasacral Parallel Shift (SSPS) technique for lumbosacral plexus blockade. Twenty-six healthy subjects aged 21–36 years received two SSPS blocks (20 mL 2% lidocaine-epinephrine [1 : 200,000] added 1 mL diluted contrast) guided by ultrasound/MRI fusion versus ultrasound. Number (proportion) of subjects with motor blockade of the femoral and obturator nerves and the lumbosacral trunk was equal (ultrasound/MRI, 23/26 [88%]; ultrasound, 23/26 [88%]; *p* = 1.00). Median (interquartile range) preparation and procedure times (s) were longer for the ultrasound/MRI fusion guided technique (686 [552–1023] versus 196 [167–228], *p* < 0.001 and 333 [254–439] versus 216 [176–294], *p* = 0.001). Both techniques produced perineural spread and corresponding sensory analgesia from L2 to S1. Epidural spread and lidocaine pharmacokinetics were similar. Different compartmentalized patterns of injectate spread were observed. Ultrasound/MRI fusion guided SSPS was equally effective and safe but required prolonged time, compared to ultrasound guided SSPS. This trial is registered with EudraCT (2013-004013-41) and ClinicalTrials.gov (NCT02593370).

## 1. Introduction

An effective, safe, and easy-to-perform peripheral nerve block technique for surgical anesthesia of the hip joint and concurrent postoperative analgesia would be advantageous, because many of the patients admitted for hip fracture are elderly, fragile, and sometimes impaired by severe cardiovascular comorbidity [1].

General and spinal anesthesia is associated with increased hemodynamic instability, anesthesia related mortality, and complications in old and multimorbid patients [2].

Compared to general and spinal anesthesia, more stable hemodynamics, fewer complications, and superior postoperative pain relief are achieved with peripheral regional anesthesia with a minimal use of opioids [3–6].

The femoral and obturator nerves are the terminal nerves of the lumbar plexus that innervate the hip joint together with the lumbosacral trunk of the sacral plexus. All these nerves can be anesthetized with a single injection paravertebrally between the transverse process of the fifth lumbar (L5) vertebra and the cranial border of the sacral ala [7, 8]. However, the accuracy of targeting the nerves with an ultrasound guided injection may be impaired due to the deep location of the target nerves as well as the lumbosacral bony structures generating acoustic shadows impeding the visibility of the needle trajectory [9], especially in old, fragile, comorbid, or obese patients [10–13]. Consequently, the efficiency and safety of the blockade may be undesirably affected and epidural spread of the injectate [9] as well as vascular, neural, or muscular injury may occur.

The accuracy of image guided procedures may be improved by fusing real-time ultrasound with magnetic resonance imaging (MRI) thus defeating the limitations of ultrasonography as a stand-alone technique [14, 15]. Furthermore, the image fusion technology includes electromagnetic needle tip tracking, which allows the operator to continuously assess the best needle insertion point, the needle trajectory, and the target of the injection. Finally, image fusion can be used to better the understanding of (ultrasonographic) anatomy and needle guidance and to refine existing ultrasound guided needle techniques [16]. Fusion of real-time ultrasound and computer tomography (CT) or MRI has been used successfully especially in interventional radiology [14, 15]. In regional anesthesia, an application of fused ultrasound/CT or MRI of the lumbar spine has been briefly described in a phantom and in volunteers, respectively, but no injections were performed [17]. In chronic pain therapy, only a few cadaver and case reports have assessed ultrasound/CT or MRI fusion guided injections primarily of the sacroiliac joint, hand, and wrist [18–21].

In this exploratory randomized controlled crossover trial, we aim to investigate real-time ultrasound/MRI fusion versus ultrasound guidance applied on the Suprasacral Parallel Shift (SSPS) technique for lumbosacral plexus blockade [22]. Primary outcome is the proportion of study subjects with motor blockade of the femoral and obturator nerves as well as the lumbosacral trunk. Secondary outcomes are procedure-related, perineural spread of injectate analyzed by MRI, epidural spread, sensory blockade, lidocaine pharmacokinetics, and cost-effectiveness. In addition, we aim to explore compartmentalized patterns of injectate spread by MRI.

## 2. Methods and Materials

### 2.1. Ethics

The Regional Research Ethics Committee (MJ: 1-10-72-179-13), the Danish Medicines Agency (2013-004013-13), and the Danish Data Protection Agency (1-16-02-160-14) approved this randomized controlled crossover trial. The study was registered in EudraCT (2013-004013-41) and in ClinicalTrials.gov (NCT02593370), monitored by the Good Clinical Practice unit at Aalborg and Aarhus University Hospitals, and complied with the Helsinki Declaration. Written informed consent was obtained from all subjects.

### 2.2. Recruitment

ASA I subjects aged ≥18 years were recruited through a Danish website for research volunteers. Subjects who were non-Danish speakers or unable to cooperate, had a history of allergy to local anesthetics or contrast agents, daily consumption of analgesics, abuse of medicine or alcohol, contraindication to MRI or infection or prior surgery of the paravertebral lumbosacral region, or who were legally incompetent were excluded.

The study was conducted at the Department of Radiology, Aarhus University Hospital, in Denmark during two three-day sessions with a one-week interim period in October to November 2015. The volunteers received payment for participation.

### 2.3. MRI for Fusion with Ultrasound

An experienced radiographer recorded supine MRI scans of all subjects with a 1.5 T Philips Ingenia MRI scanner (Koninklijke Philips Electronics NV, Eindhoven, Netherlands) upon arrival on the first session. The subjects were scanned with a pillow under their knees to minimise lumbar lordosis and a dS flex coverage anterior coil for signal reception. The recordings of the lumbar spine were coronal 3D T2-TSE sequences with an scanning resolution of 1.00 × 1.00 × 2.00 mm^3^ (overlapping 2.40 mm slices, 1.20 spacing), TE 60 ms, and TR 1200 ms. A feet-head phase encoding was applied to minimise artifacts due to respiration and peristalsis. All sequences were reconstructed to 0.78 × 0.78 × 1.00 mm^3^ resolution and converted to axial orientation using OsiriX v6.5.2 64-bit (Pixmeo SARL, Bernex, Switzerland) prior to upload to the ultrasound system with image fusion software (Epiq 7 1.4; Koninklijke Philips Electronics NV, Eindhoven, Netherlands), because the system only accepts axially oriented datasets for fusion.

### 2.4. Lumbosacral Plexus Block Procedure

The subjects were monitored with three-lead ECG, noninvasive blood pressure measurement, and pulse oximetry. Peripheral intravenous access was established for blood sampling and safety.

All blocks were performed with the Epiq 7 1.4 ultrasound system. The regional anesthetist (TFB) who performed all blocks has extensive clinical experience with ultrasound and electrical nerve stimulation guided nerve blocks and experimental experience with ultrasound/MRI fusion guided lumbosacral procedures.

While performing all blocks, the field generator (Koninklijke Philips Electronics NV, Eindhoven, Netherlands) was positioned over the lumbosacral region to generate the electromagnetic field necessary for fusion or to strengthen blinding of the subjects. After prescanning and any coregistration of ultrasound and MRI, the skin was swapped with chlorhexidine in isopropyl alcohol and covered with a sterile fenestrated drape. The curved array ultrasound probe (C5-1; Koninklijke Philips Electronics NV, Eindhoven, Netherlands) with the attached sensor was draped with a sterile cover. The skin and subcutaneous tissue were infiltrated with 2 mL 2% lidocaine prior to insertion of a 22 Gauge, 100 mm nerve block needle (Stimuplex Ultra; B. Braun, Melsungen, Germany). The injectate of each nerve block was 20 mL 2% lidocaine-epinephrine [1 : 200,000] with 1 mL diluted MRI contrast (0.13 mL 27.9% gadoterate meglumine [Dotarem®; Guerbet, Roissy CdG Cedex, France] and 0.87 mL 0.9% isotonic saline) added.

**Figure 1:**
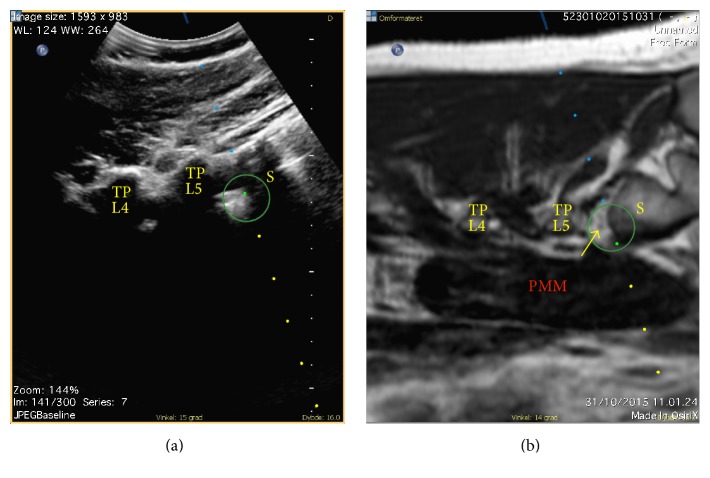
The ultrasonographic (a) and MR (b) images are fused and displayed side-by-side. The blue line in the top is the projection of the block needle and the large green circle marks the anticipated intersection of the block needle tip and the ultrasound beam, here coinciding with the rami of spinal nerve L5 (yellow arrow) displayed on the MR image. The line of small blue and yellow dots marks the anticipated trajectory of the block needle prior to and after the intersection with the ultrasound beam, respectively. PMM, major psoas muscle; S, sacral ala; TP L4-5, transverse processes of the fourth and fifth lumbar vertebral bodies.

*Ultrasound/MRI Fusion Guided SSPS*. The subject was placed supine. The patient tracker, serving as a reference for the sensors mounted on the probe and the block needle, was affixed to the subject's iliac crest with adhesive tape on the side to be anesthetized. The probe was oriented axially on the abdomen. An identical reference point in the axial plane at the bifurcation of the common iliac arteries was used for coregistration of the real-time ultrasound and the MRI dataset. After coregistration, the ultrasonographic and MR images moved synchronically with any movement of the probe. The images were aligned using the iliac arteries, the aortic bifurcation, and the anterior margin of the lumbar vertebral body at the same level. Next, the subject was turned to the lateral decubitus position with the side to be anesthetized facing upwards. In order to take account for any misalignment due to the position change, the alignment of the fused datasets was fine adjusted using the borders of the L5 transverse process and vertebral body as well as the positions of the psoas major, quadratus lumborum, and erector spinae muscles. The probe was placed in the sagittal plane across the caudal border of the transverse process of L5 and the cranial border of the sacral ala, visualizing the interspace (osteofibrotic tunnel) in-between the bony structures on both images. Based on ultrasound/MRI visualization of the intertransverse ligament (posteriorly) and the lumbosacral ligament (anteriorly) marking out the osteofibrotic tunnel, the tip of the block needle was placed in the anticipated position for needle insertion caudad to the intercristal line. Using needle navigation, the position and angle of the insertion were adjusted until the anticipated intersection of the needle tip and the ultrasound beam coincided with the target compartment posterior to the psoas major muscle [8, 9] displayed on the MR image (Figure 1). Guided by real-time ultrasound/MRI fusion and needle navigation, the needle was advanced with an out-of-plane technique until a “loss of resistance” confirmed the visualized penetration through the lumbosacral ligament and the needle tip position anterior to the ligament on MRI.

*Ultrasound Guided SSPS*. This technique was performed in the lateral decubitus position and has been described in-depth previously [9]. The endpoint of injection was “loss of resistance” confirming the needle penetration of the lumbosacral ligament, sonographically visualized if possible.

An electrical nerve stimulator (0.1 ms, 2 Hz, 0.2 mA) was connected to the block needle during both procedures in order to decrease the risk of intraneural injection of local anesthetics. Prior to injection, any response to electrical nerve stimulation with 0.3 to 0.5 mA was registered [23]. The electrical nerve stimulation was use for safety only, not needle guidance. The local anesthetic with contrast was injected with intermittent aspiration.

Time zero (*T*_0_) min was the time of withdrawal of the block needle from the skin after completed injection. All subjects were followed up until *T*_90_ for data sampling and were observed for adverse effects until the sensorimotor blockade had worn off.

### 2.5. Outcomes and Assessment

The primary outcome was the proportion of subjects with motor blockade of the femoral and obturator nerves as well as the lumbosacral trunk. Motor blockade was defined as ≥1 N reduction in muscle force (N) of the knee extensors, hip adductors, and hip abductors, respectively, at *T*_40_ compared to baseline. Muscle force was estimated in the supine position with a dynamometer (Commander Muscle Testing; JTECH Medical, Midvale, USA) maintained immobile by a steady grip of an observer. The observer instructed the subject to exert maximal pressure against the dynamometer during knee extension (with 90° flexion of the hip and knee joints), hip adduction (with extended and 45° abducted lower limb), and hip abduction (with extended lower limb). The highest value of three tests with 20 s intermittent intervals was recorded for each motion.

The secondary outcomes were (a) preparation time (s) from positioning of the subject on the bed until end of prescanning and coregistration, if any; (b) block procedure time (s) from placement of the probe on the skin until withdrawal of the block needle after completed injection; (c) number of needle insertions defined as each withdrawal of the needle followed by an advancement regardless the number of skin penetrations; (d) needle insertion point defined as the horizontal distance (cm) from the median to the skin penetration; (e) depth of needle tip gauged by reading the distance (cm) marked on the needle shaft at the endpoint of the injection; (f) minimal electrical nerve stimulation (mA) required to trigger any sensorimotor response immediately prior to injection; (g) type of response to electrical nerve stimulation (“Quadriceps,” “Adductor,” “Other motor,” “Paresthesia,” and “None”); (h) maximum procedural discomfort assessed by the subject on a numeric rating scale (NRS, 0 = “no discomfort”, 10 = “worst possible discomfort”) at *T*_0_; (i) change in mean arterial blood pressure (ΔMAP) from baseline to *T*_5_; (j) perineural injectate spread; (k) epidural injectate spread; (l) sensory blockade; (m) maximum plasma concentration of lidocaine (*C*_max_ of p-lidocaine, *μ*g/mL); (n) time to *C*_max_ (*T*_omc_) of p-lidocaine (min); (o) p-lidocaine concentration-time area under the curve; and (p) cost-effectiveness.

Injectate spread was analyzed on axial 3D T1-weighted MRI sequences (mDixonAll generating in-phase, out-of-phase, water and fat images as well as diffusion weighted images) sampled with a Philips Achieva 3.0 T dstream scanner (Koninklijke Philips Electronics, Eindhoven, Netherlands) at *T*_15_. Perineural spread was assessed for the anterior rami of spinal nerves L2-S1, the femoral, obturator, and lateral femoral cutaneous nerves as well as the lumbosacral trunk. Perineural spread was considered “present” when direct contact between the injectate and the target nerve was visualized.

As an exploratory analysis, we observed different patterns of confinement of injectate inside the fascial compartments medial, posterior, and lateral to the psoas major muscle, respectively, as well as associated spread of injectate around compartment-specific nerves.

Epidural spread was considered “present” when there was circumferential epidural distribution of the injectate on any axial MRI level and concomitant bilateral blockade of cold in at least one pair of dermatomes.

Sensory blockade of cold, warmth, touch, and pain of the dermatomes Th12-S3 [24] and the skin innervated by the lateral femoral cutaneous nerve was tested with standardized stimuli (25° and 40° thermo test [Rolltemp II; Somedic, Hörby, Sweden], brush [SENSELab™ Brush-05; Somedic AB, Hörby, Sweden], and punctuated pin prick [PinPrick 512 mN; MRC Systems GmbH, Heidelberg, Germany]) at *T*_50_. Sensation for each stimulus was assessed as “present” or “reduced/absent,” where “reduced/absent” was considered a successful sensory blockade. The dermatomes Th12, L1, S2, and S3 were included in order to assess the effect of any epidural spread.

For the analysis of p-lidocaine, blood samples were collected at *T*_0,5,10,20,40,60,  and  90_ and centrifuged at 1,800*g* for 9 min. The plasma was transferred to 1.5 mL cryotubes and stored at −80°C until analysis with liquid chromatography tandem mass spectrometry [25].

The difference in mean marginal cost of the interventions was calculated as a measure of cost-effectiveness (extra price per patient) [26]. Unit costs were collected in Danish Kroner (DKK) in July 2016 and converted into US dollars (GBP/euros) in October 2016 (100 DKK = $14.86 [£12.08/€13.44]). Average annual total wages were used to calculate unit costs for time spent by medical staff. Because of the complexity of calculating the expense for the 1.5 T MRI scanner use, this cost is given as a time unit.

### 2.6. Randomization and Blinding

JMCS enrolled all subjects. Two study-independent assistants randomly allocated 26 consecutive subject identification numbers to sequences of interventions (Ultrasound/MRI fusion guided SSPS on day one and ultrasound guided SSPS on day two or vice versa) and side (right on day one and left on day two or vice versa). Twenty-six sheets with the sequences preprinted were put in 26 identical opaque and sealed envelopes marked 1 to 26. TFB and SB double-checked the allocated intervention and side immediately prior to each procedure without revealing it to others. The sheet was reenveloped and resealed. The procedure was repeated prior to the second intervention.

All sampling and analyses of data were blinded to the intervention. All interventions were performed with identical trial setup and equipment in order to blind the subjects. The MRI records of injectate spread were anonymised by a radiographer and hereafter analyzed in a random order by TFB.

### 2.7. Statistics

The primary outcome was proportion of subjects with motor blockade of the femoral and obturator nerves as well as the lumbosacral trunk. We hypothesized an increase in the proportion from 75% with ultrasound guidance to 100% with ultrasound/MRI fusion guidance. Detection of a 25% increase with 80% power (1 − *β*) and *α* = 0.05 would require a sample size of 24 subjects in a two-sided crossover analysis [27]. To avoid decreased power due to dropouts, we included 26 subjects.

Statistics were analyzed with Stata IC 14 (StataCorp LP, College Station, USA). Normality of distribution was assessed visually with the normal Q-Q-plot. Normally distributed differences between paired continuous variables were analyzed with one-sample Student's *t*-test. Nonnormally distributed differences between paired continuous variables and differences between paired ordinal variables were analyzed with Wilcoxon matched-pairs signed rank test. Differences between paired categorical variables were analyzed with McNemar's test. The level of significance was 0.05. Data are presented as mean (standard deviation [SD]) for continuous variables with normal distribution, as median (interquartile range [IQR] [range]) for continuous variables with nonnormal distribution and ordinal variables, and as number (proportion) for categorical variables.

## 3. Results

**Figure 2:**
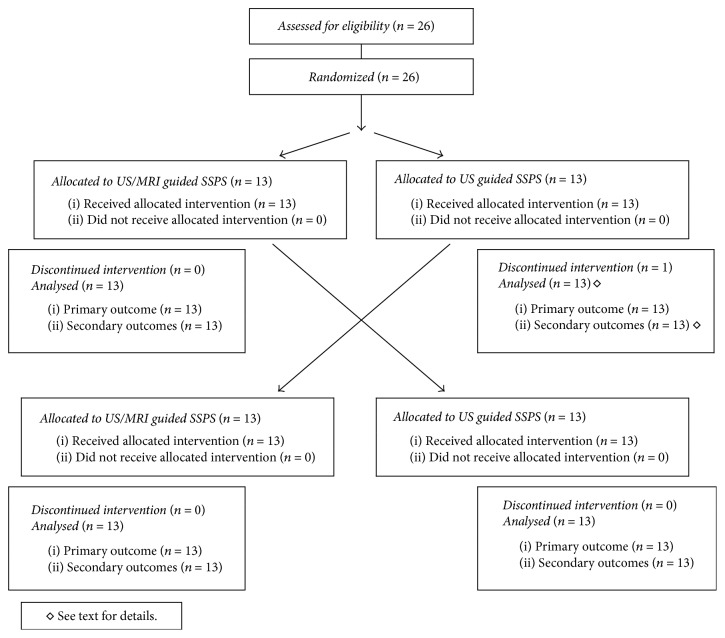
Modified CONSORT 2010 flow diagram of the study subjects receiving ultrasound (US)/magnetic resonance imaging (MRI) fusion versus US guided lumbosacral plexus blockade with the Suprasacral Parallel Shift (SSPS) technique.

Twenty-six subjects (14/26 [54%] males) were enrolled during October 3 to 24, 2015 (Figure 2). Twenty-five subjects completed both interventions and follow-up per protocol. One ultrasound guided SSPS intervention was aborted due to aspiration of blood, but the subject completed the follow-up and contributed with his data per protocol.

The median (IQR [range]) age for all 26 subjects was 22 (22–24 [21–36]) years, mean (SD) weight was 73.2 (11.7) kg, mean (SD) height was 178 (8.1) cm, and mean (SD) BMI was 23.4 (2.7) kg·m^−2^.

The number (proportion) of subjects with motor blockade of the femoral and obturator nerves as well as the lumbosacral trunk was equal (ultrasound/MRI, 23/26 [88%]; ultrasound, 23/26 [88%]; *p* = 1.00).  Table 1 displays the underlying data on muscle force and the number (proportion) of motor blockade of the femoral nerve, obturator nerve, and lumbosacral trunk, respectively.

Table 2 displays the values of the procedure-related outcomes.

There was no evidence for any difference in perineural spread to the anterior rami of spinal nerves L2 (ultrasound/MRI, 14/26 [54%]; ultrasound, 11/26 [42%]; *p* = 0.58), L3 (ultrasound/MRI, 21/26 [81%]; ultrasound, 21/26 [81%]; *p* = 1.00), L4 (ultrasound/MRI, 22/26 [85%]; ultrasound, 25/26 [96%]; *p* = 0.38), L5 (ultrasound/MRI, 10/26 [38%]; ultrasound, 18/26 [69%]; *p* = 0.057), and S1 (ultrasound/MRI, 5/26 [19%]; ultrasound, 8/26 [31%]; *p* = 0.55), the femoral (ultrasound/MRI, 16/26 [62%]; ultrasound, 13/26 [50%]; *p* = 0.61), obturator (ultrasound/MRI, 14/26 [73%]; ultrasound, 11/26 [85%]; *p* = 0.58), and lateral femoral cutaneous (ultrasound/MRI, 16/26 [62%]; ultrasound, 11/26 [42%]; *p* = 0.58) nerves, or the lumbosacral trunk (ultrasound/MRI, 10/26 [38%]; ultrasound, 15/26 [58%]; *p* = 0.58).

**Figure 3:**
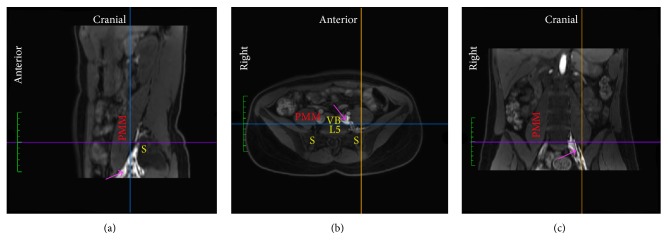
MRI of one subject visualizing spread of lidocaine-epinephrine added diluted contrast (magenta arrow) primarily medial to the psoas major muscle (PMM), that is, in the* parapsoas compartment*. (a) Sagittal plane. (b) Axial plane. (c) Coronal plane. Line (blue), position of coronal plane; Line (orange), position of sagittal plane; Line (purple), position of axial plane; S, sacral ala; VB L5, fifth lumbar vertebral body.

**Figure 4:**
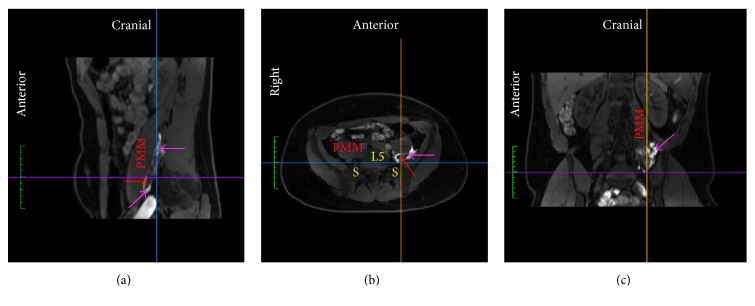
MRI of one subject visualizing spread of lidocaine-epinephrine added diluted contrast (magenta arrow) primarily posterior to the psoas major muscle (PPM), that is, in the* retropsoas subcompartment*, with minor seeping into the fascial plane between the anterior and posterior (red arrow) lamina of the PMM that contains the lumbar plexus. (a) Sagittal plane. (b) Axial plane. (c) Coronal plane. L5, fifth lumbar vertebral body; Line (blue), position of coronal plane; Line (orange), position of sagittal plane; Line (purple), position of axial plane; S, sacral ala.

**Figure 5:**
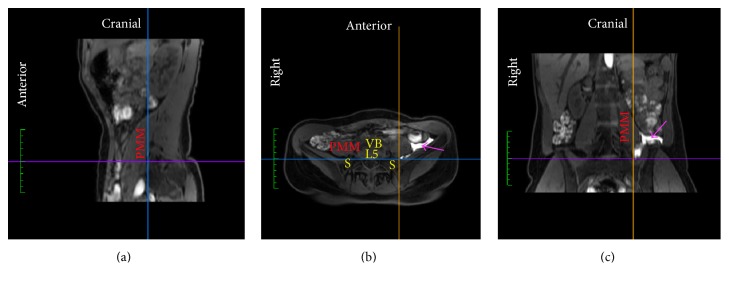
MRI of one subject visualizing spread of lidocaine-epinephrine added diluted contrast (magenta arrow) primarily lateral to the psoas major muscle (PMM), that is, in the* retroperitoneal compartment*, with minor seeping into the retropsoas subcompartment. (a) Sagittal plane. (b) Axial plane. (c) Coronal plane. Line (blue), position of coronal plane; Line (orange), position of sagittal plane; Line (purple), position of axial plane; S, sacral ala; VB L5, fifth lumbar vertebral body.

We identified characteristic patterns of compartmentalized injectate spread inside three compartments (Figures 3, 4 and 5).

No difference in compartmentalized spread of injectate was observed between the two study groups. The frequencies of compartmentalized spread of injectate in the entire population of volunteers were 27% into the* parapsoas compartment (PPC)* and* retropsoas subcompartment (RPSC)*, 27% into the PPC, 37% into the RPSC, and 9% into the* retroperitoneal compartment (RC)*. Seeping of injectate into the intrapsoas compartment, that is, the compartment between the anterior and posterior lamina of the psoas major muscle, and to the L2–L4 part of the lumbar plexus occurred in 89% of RPSC injection, 50% of PPC injection, and 40% of RC injection.

Table 3 displays the values of sensory blockade of the dermatomes Th12-S3 and the lateral femoral cutaneous nerve.

There was no evidence for any difference in epidural spread (ultrasound/MRI 3/26 (12%) subjects; ultrasound, 5/26 (19%) subjects; *p* = 0.73). The sensory effect was observed in the dermatomes L1 to S3 with individual variation.

**Figure 6:**
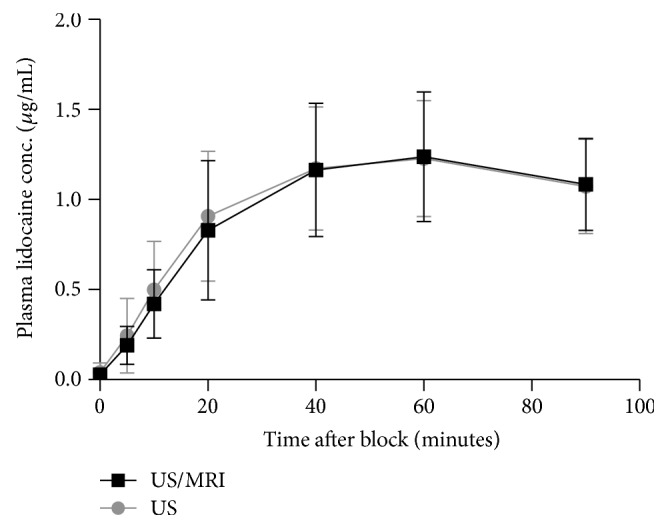
Plasma concentration of lidocaine 0 to 90 min after injection with the ultrasound/MRI fusion guided (US/MRI) versus the ultrasound guided (US) Suprasacral Parallel Shift technique for lumbosacral plexus blockade (*n* = 25). Values are presented as mean (SD).

Figure 6 illustrates the mean (SD) *C*_max_ of p-lidocaine. There was no evidence for any difference in mean (SD) *C*_max_, median (IQR [range]) *T*_omc_, or mean (SD) concentration-time area under the curve (Figure 6). One subject in the ultrasound group was excluded from the analysis due to insufficient blood sampling.

The mean marginal cost of a SSPS block was Δ/$28.19 (£22.91/€23.60) and 6 min and 34 s in the 1.5 T MRI scanner for the ultrasound/MRI fusion guided procedure compared to the ultrasound guided.

No serious adverse events were observed. One subject experienced a transitory hot flush prior to the intervention due to vasovagal needle phobia. Four subjects had two incidents of vasovagal syncope and three incidents of dizziness; two were related to reinsertion of an intravenous catheter or blood sampling during the follow-up; and one was related to previously diagnosed orthostatic hypotension.

## 4. Discussion

This is the first randomized controlled trial investigating ultrasound/MRI fusion guided lumbosacral plexus blockade. We found that the ultrasound/MRI fusion guided technique was equally effective and safe, but required longer preparation and block procedure time compared to the ultrasound guided technique.

### 4.1. Block Success

The initial hypothesis of a higher proportion of subjects with motor blockade of the femoral and obturator nerves as well as the lumbosacral trunk with ultrasound/MRI fusion guidance compared to ultrasound guidance was falsified. This may be explained by the demographics of the study subjects. That is, the target clinical group would be elderly and fragile patients, in whom the ultrasonoanatomical image quality may be impaired and in whom additional MRI visualization and needle navigation with fusion might improve the efficiency of needle guidance. In young healthy normal-weighted volunteers, however, adequate ultrasonographic quality is achieved with a higher frequency thus making the MRI scan, image fusion, and needle navigation redundant. Nonetheless, we assessed the fusion guided technique in healthy volunteers instead of clinical patients, because only ultrasound/MRI fusion of the lumbar spine, and not fusion guided lumbar needle insertions, has been briefly described in phantoms and volunteers before [17]. Furthermore, the body position of the subjects was different during the MRI sampling and the ultrasound/MRI fusion guided needle procedure. This may have affected the topography and dimensional stability of the anatomical structures under study, and hence the accuracy of the ultrasound/MRI fusion guided injection [15, 19]. However, we sampled the MRI dataset supine because this is technically and clinically most optimal. Moreover, any misalignment can be manually adjusted and a pilot study revealed no evidence that the target nerves, situated paravertebral to the rigid lumbar spine, moved significantly during change from supine to lateral decubitus. Yet, we cannot fully rule out such an effect.

### 4.2. Block Procedure-Related Outcomes

The prolonged time for the ultrasound/MRI fusion guided technique is in keeping with previous studies concerning real-time fusion [14]. It is explained by the extra time spent on coregistration and alignment of the datasets and on needle navigation. It is also reflected by the higher mean marginal cost and prolonged 1.5 T MRI time use of the fusion guided technique. Notably, both success rate and procedure time of a new technique follow a learning curve and technical perfection requires practice [14].

### 4.3. MRI Analysis of Injectate Spread versus Sensorimotor Blockade

We observed spread of injectate inside three fascial compartments. The first compartment is medial to the psoas major muscle and the iliopsoas compartment [28] (Figure 3). We therefore suggest referring to this as the* parapsoas compartment (PPC)*. We observed that the PPC extends from the level of the neural foramen of vertebra L4 cranially to the neural foramen of S1 caudally. Caudal to the transverse process of L5, the iliopsoas fascia is tied down to the sacral ala and separates the PPC from the iliopsoas compartment [28]. The PPC contains the anterior rami of spinal nerves L4-S1, the lumbosacral trunk, and the obturator nerve. The second compartment is a triangular groove that extends from the transverse process of L5 and the iliolumbar ligament cranially and between the psoas major and iliac muscles until they become fused as the iliopsoas muscle caudally (Figure 4). The compartment is bounded by the iliopsoas fascia, which medially separates it from the PPC and laterally covers the groove between the psoas major and iliacus muscles. It has been described previously [29], however, as it is a subcompartment of the iliopsoas compartment, we suggest referring to this as the* retropsoas subcompartment (RPSC).* The RPSC contains the femoral and lateral femoral cutaneous nerves, as they emerge from the posterolateral border of the psoas major muscle caudal to the level of the iliac crest. The third compartment is the retroperitoneal fat-pad compartment between the peritoneum and the transversalis fascia and lateral to the iliopsoas compartment (Figure 5). We suggest referring to this as the* retroperitoneal compartment (RC)*. The RC contains none of the major terminal lumbar plexus nerves.

In all subjects, the injectate spread primarily into one or two of the three identified compartments. Hence the injectate primarily spread perineurally around the anterior rami of spinal nerves L4-S1, the lumbosacral trunk and the obturator nerve inside the PPC, around the femoral and lateral femoral cutaneous nerves inside the RPSC, or around no nerves inside the RC. Injectate spread inside the PPC therefore resulted in increased sensorimotor blockade of primarily the anterior rami of spinal nerves L4-S1, the lumbosacral trunk (superior gluteal nerves), and the obturator nerve, while injectate spread inside the RPSC resulted in increased sensorimotor blockade of primarily the femoral and lateral femoral cutaneous nerves. Injectate spread inside the RC had no sensorimotor effect since the compartment contains no major terminal nerves of the lumbosacral plexus.

Few previous studies have compared ultrasonography and MRI of the lumbosacral anatomy [30] and analyzed injectate spread with MRI [9, 31, 32]. The sensory mapping demonstrated segmental anesthesia from L2 to S1 in accordance with the perineural spread analyzed by MRI. A cadaver study on lumbosacral plexus blockade guided by anatomical landmarks showed weak staining of spinal nerve S1 in only 3/20 (15%) cadavers [7]. In contrast, our study demonstrates that an injection at the neuraxial level of L5/S1 may indeed block the cranial part of the sacral plexus.

Nonetheless, sensory mapping of dermatomes should be interpreted with caution because it may be unreliable due to anatomical variation and overlapping of innervation of adjacent cutaneous segments and terminal nerves territories [33]. We used motor blockade as a surrogate of sensory blockade of the femoral nerve, obturator nerve, and the lumbosacral trunk. However, the motor blockade definition does not take account for bi- and triple nerve innervation of specific muscle groups or measurement error of the method to estimate muscle force. Because knowledge concerning the correlation between reduced muscle force in healthy volunteers and sufficient motor blockade in clinical patients is sparse, the values of block success should be considered as a measure of comparison of the techniques, not as a clinically applicable measure. Due to this complexity, we recommend inclusion of an objective analysis such as MRI recordings of injectate spread when validating new techniques in healthy volunteers.

### 4.4. Compartmentalized Injectate Spread and a Theoretical Ultrasound/MRI Fusion Guided Anterior Approach

**Figure 7:**
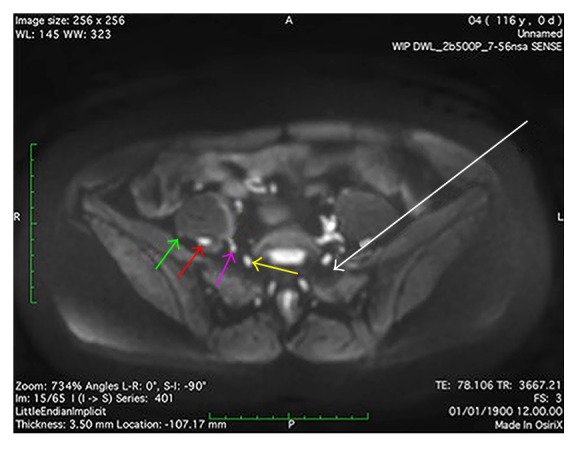
Axial diffusion weighted MRI at the level of the cranial border of the sacral ala, demonstrating a possible anterior approach to the lumbosacral plexus in the supine position. A needle (white arrow) can be inserted close to the anterior superior iliac spine and advanced between the psoas major and iliacus muscles. A first injection of local anesthetic into the* retropsoas subcompartment* will spread to the femoral nerve (red arrow) and the lateral femoral cutaneous nerve (green arrow). A second injection of local anesthetic into the* parapsoas compartment* will spread to the anterior rami of spinal nerves L4 and L5, the lumbosacral trunk (yellow arrow) and the obturator nerve (pink arrow).

**Figure 8:**
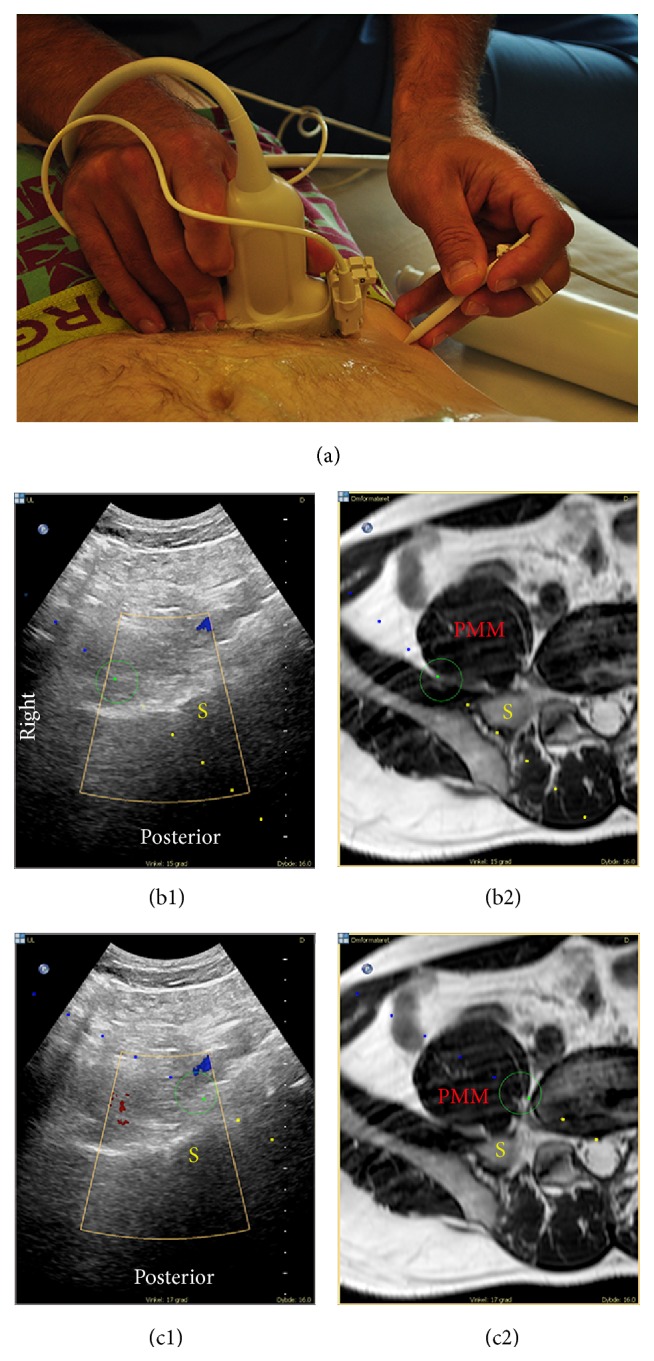
The anterior lumbosacral plexus approach guided by real-time ultrasound/MRI fusion in an anticipated experimental setting. (a) The probe is axially oriented, slightly rotated clock-wise, and medial to the anterior superior iliac spine where the phantom needle is oriented in-plane with the US/MR image planes. (b) Fused real-time ultrasound (b1) and MRI (b2) depicting the needle trajectory into the* retropsoas subcompartment*. (c) Fused real-time ultrasound (c1) and MRI (c2) depicting the needle trajectory through the psoas major muscle (PMM) into the* parapsoas compartment*. Guided by real-time ultrasound/MRI fusion and needle navigation, the “insertion point” and angulation of the phantom needle are adjusted until the anticipated intersection between the needle tip and the ultrasound beam (green circle) coincides with the target lumbosacral plexus nerves in the retropsoas compartment (b) and in the parapsoas subcompartment (c) anterior to the border of the sacral ala (S). The line of small blue and yellow dots marks the anticipated trajectory of the block needle prior to and after the intersection with the ultrasound beam, respectively.

The MRI recordings allow analysis of patterns of injectate spread, which together with high-resolution MRI for fusion with real-time ultrasound offer the potential of improved understanding of the (ultrasonographic) anatomy [16]. In the present study, we identified characteristic patterns of injectate spread in the different fascial compartments. The observed patterns of spread imply that local anesthetic has to be injected into the PPC as well as the RPSC for sufficient spread to all target nerves relevant for anesthesia of the hip joint with a high clinical success rate. However, the visualization of the PPC and RPSC is impeded by bony structures when the ultrasound guided SSPS technique is employed. Furthermore, in the lateral decubitus position with the side to be anesthetized facing upwards, gravity may facilitate medial spread of the local anesthetic to the epidural space [34]. An ultrasound/MRI fusion guided anterior technique in the supine position may overcome these limitations and supply a safe, efficient, and easy needle path from the skin surface to the target nerves inside the PPC as well as the RPSC (Figures 7 and 8).

### 4.5. Pharmacokinetics of p-Lidocaine

The mean *C*_max_ of p-lidocaine was similar for the techniques and peaked approximately one hour after injection. This is in keeping with previous studies investigating plasma concentration of local anesthetics in regional anesthesia [9, 32, 35]. No dose-finding studies have been conducted for the SSPS technique, but the minimal effective anesthetic volume of 0.5% ropivacaine to accomplish a successful Shamrock lumbar plexus blockade in 95% of patients (ED_95_) is 36.0 mL (95% CI 19.7 to 52.2) [36]. Because the aim of this study was to compare two techniques in a standardized setting, and not to achieve maximum block success, and the subjects were discharged on the same day, we chose a comparatively low dose of 20 mL 2% lidocaine-epinephrine corresponding approximately to the ED_50_ of 0.5% ropivacaine [36] and allowing fast discharge. However, injection of more clinical relevant local anesthetic volumes in excess of 20 mL would result in increased *C*_max_. Also, pharmacokinetics of local anesthetic changes with age [37]. The present results might therefore not be directly applicable in elderly patients.

### 4.6. Limitations

Apart from the external limitations above, the expert anesthetist performing all blocks could not be blinded, as is the case with all procedure-related studies. In order to limit this source of bias, we adhered to a strict double-controlled protocol.

### 4.7. Perspectives

Ultrasound/MRI fusion for needle guidance in the lumbosacral region is an evolving technique and is proven to be neither more inaccurate nor more unsafe compared to ultrasound guidance. Future studies of real-time ultrasound/MRI needle guidance may include automatic coregistration based on image recognition or MRI-compatible external fiducials, or techniques that minimise the effect of position change and thereby improving the accuracy, time-efficiency, and ease-of-performance.

### 4.8. Conclusion

The ultrasound/MRI fusion guided SSPS technique was equally effective and safe but required longer time, compared to the ultrasound guided SSPS technique. Three patterns of compartmentalized injectate spread indicate that local anesthetic has to be injected into the parapsoas compartment as well as the retropsoas subcompartment to block the lumbosacral nerves innervating the hip joint.

## Figures and Tables

**Table 1:** Baseline and postblock muscle force as well as number of subjects with motor blockade of the femoral nerve, obturator nerve, and the lumbosacral trunk for ultrasound/MRI fusion guided versus ultrasound guided lumbosacral plexus blockade with the Suprasacral Parallel Shift technique. Values are displayed as median (IQR [range]) or number (proportion).

	US^*∗*^/MRI^†^	US^†^
	(*n* = 26)	(*n* = 26)
*Femoral nerve (knee extension)*		
Baseline muscle force; N	244 (204–266 [176–343])	229 (215–253 [136–374])
Postblock muscle force; N	75 (0–121 [0–244])	72 (0–134 [0–255])
Motor blockade	25 (96%)	24 (92%)
*Obturator nerve (hip adduction)*		
Baseline muscle force; N	138 (114–176 [105–255])	134 (114–176 [101–237])
Postblock muscle force; N	0 (0–70 [0–149])	0 (0–31 [0–209])
Motor blockade	25 (96%)	24 (92%)
*Lumbosacral trunk (hip abduction)*		
Baseline muscle force; N	147 (114–160 [79-204])	144 (114–167 [79–233])
Postblock muscle force; N	79 (35–105 [0–173])	54 (41–79 [0–169])
Motor blockade	24 (92%)	24 (92%)

Motor blockade was defined as a decrease in postblock muscle force compared to baseline.

^*∗*^US: ultrasound.

^†^MRI: magnetic resonance imaging.

**Table 2:** Procedure-related outcomes for ultrasound/MRI fusion guided versus ultrasound guided lumbosacral plexus blockade with the Suprasacral Parallel Shift technique. Values are displayed as median (IQR [range]), number (proportion), or mean (SD).

	US^*∗*^/MRI^†^	US	*p*
	(*n* = 26)	(*n* = 26)	
Preparation time; s	686 (552–1023 [393–2501])	196 (167–228 [105–351])	<0.001
Block procedure time; s	333 (254–439 [201–1421])	216 (176–294 [117–458])	0.001
Number of needle insertions	4.5 (3.0–7.0 [2.0–24.0])	5.0 (3.0–7.0 [2.0–15.0])	0.87
Needle insertion point from midline; cm	4.0 (4.0–5.0 [2.0–6.0])	6.0 (5.0–6.0 [4.0–8.0])	<0.001
Needle depth; cm	8.0 (7.0–9.0 [5.0–10.0])	8.0 (7.0–8.5 [4.0–10.0])	0.37
Minimal nerve stimulation; mA	0.50 (0.50–0.50 [0.20–0.60])	0.50 (0.40–0.50 [0.30–0.50])	0.075
Electrical nerve stimulation response			0.37
1 Quadriceps femoris	4 (15%)	4 (15%)	1.00
2 Adductor	0 (0%)	1 (4%)	1.00
3 Other motor	0 (0%)	0 (0%)	1.00
4 Paresthesia	2 (8%)	0 (0%)	0.50
0 None	20 (77%)	21 (81%)	1.00
Procedural discomfort; NRS 0–10^‡^	2 (1–3 [0–7])	3 (2–4 [0–5])	0.036
ΔMAP; mmHg^§^	0.23 (12.77)	−4.50 (10.44)	0.070

^**∗**^US: ultrasound.

^†^MRI: magnetic resonance imaging.

^‡^NRS: numeric rating scale.

^§^ΔMAP: change in mean arterial pressure from baseline to 5 min after completed injection of local anesthetic.

**Table 3:** Number of subjects with sensory blockade of the dermatomes Th12-S3 and the skin area innervated by the lateral femoral cutaneous nerve after ultrasound/MRI fusion guided (*n* = 26) versus ultrasound guided (*n* = 26) lumbosacral plexus blockade with the Suprasacral Parallel Shift technique. Values are displayed as number (proportion).

	Cold			Warmth			Touch			Pain		
	US^*∗*^/MRI^†^	US	*p*	US/MRI	US	*p*	US/MRI	US	*p*	US/MRI	US	*p*
Th12	2 (8%)	0 (0%)	0.50	1 (4%)	1 (4%)	1.00	4 (15%)	1 (4%)	0.38	7 (8%)	2 (8%)	1.00
L1	4 (15%)	3 (12%)	1.00	2 (12%)	4 (15%)	1.00	9 (35%)	6 (23%)	0.55	7 (27%)	4 (15%)	0.51
L2	8 (31%)	10 (38%)	0.79	9 (35%)	10 (38%)	1.00	9 (35%)	11 (42%)	0.80	12 (46%)	11 (42%)	1.00
L3	18 (69%)	16 (62%)	0.75	13 (50%)	16 (42%)	0.58	7 (27%)	9 (35%)	0.75	15 (58%)	9 (35%)	0.18
L4	18 (69%)	15 (58%)	0.61	17 (65%)	14 (54%)	0.61	13 (50%)	13 (50%)	1.00	13 (50%)	14 (54%)	1.00
L5	10 (38%)	11 (42%)	1.00	9 (35%)	12 (46%)	0.55	8 (31%)	10 (38%)	0.75	8 (31%)	10 (38%)	0.75
S1	16 (62%)	14 (54%)	0.63	16 (52%)	18 (69%)	0.69	3 (12%)	10 (38%)	0.016	8 (31%)	12 (46%)	0.39
S2	5 (19%)	7 (27%)	0.75	7 (27%)	10 (38%)	0.55	8 (31%)	7 (27%)	1.00	7 (27%)	6 (23%)	1.00
S3	5 (19%)	8 (27%)	0.75	5 (19%)	7 (27%)	0.75	7 (27%)	9 (25%)	0.77	7 (27%)	8 (31%)	1.00
LFCN^‡^	13 (50%)	9 (35%)	0.39	14 (54%)	10 (38%)	0.34	16 (62%)	10 (38%)	0.18	17 (65%)	10 (38%)	0.092

^**∗**^US: ultrasound.

^†^MRI: magnetic resonance imaging.

^‡^LFCN: lateral femoral cutaneous nerve.
